# Exploring evolution and diversity of Chinese Dipterocarpaceae using next-generation sequencing

**DOI:** 10.1038/s41598-019-48240-y

**Published:** 2019-08-12

**Authors:** Tijana Cvetković, Damien Daniel Hinsinger, Joeri Sergej Strijk

**Affiliations:** 1 Biodiversity Genomics Team, Plant Ecophysiology & Evolution Group, Guangxi Key Laboratory of Forest Ecology and Conservation, College of Forestry, Daxuedonglu 100, Nanning, Guangxi 530005 P.R. China; 2 State Key Laboratory for Conservation and Utilization of Subtropical Agro-bioresources, College of Forestry, Guangxi University, Nanning, Guangxi 530005 P.R. China; 3 Alliance for Conservation Tree Genomics, Pha Tad Ke Botanical Garden, PO Box 959, 06000 Luang Prabang, Lao PDR

**Keywords:** Molecular evolution, Phylogenetics

## Abstract

Tropical forests, a key-category of land ecosystems, are faced with the world’s highest levels of habitat conversion and associated biodiversity loss. In tropical Asia, Dipterocarpaceae are one of the economically and ecologically most important tree families, but their genomic diversity and evolution remain understudied, hampered by a lack of available genetic resources. Southern China represents the northern limit for Dipterocarpaceae, and thus changes in habitat ecology, community composition and adaptability to climatic conditions are of particular interest in this group. Phylogenomics is a tool for exploring both biodiversity and evolutionary relationships through space and time using plastome, nuclear and mitochondrial genome. We generated full plastome and Nuclear Ribosomal Cistron (NRC) data for Chinese Dipterocarpaceae species as a first step to improve our understanding of their ecology and evolutionary relationships. We generated the plastome of *Dipterocarpus turbinatus*, the species with the widest distribution using it as a baseline for comparisons with other taxa. Results showed low level of genomic diversity among analysed range-edge species, and different evolutionary history of the incongruent NRC and plastome data. Genomic resources provided in this study will serve as a starting point for future studies on conservation and sustainable use of these dominant forest taxa, phylogenomics and evolutionary studies.

## Introduction

Forests hold more than 75 percent of the world’s terrestrial biodiversity and provide products and services for socioeconomic development of millions of people^1^. Over the course of the last few centuries, over a billion hectares of forest lands have been degraded or deforested, which underlie broad scale climate changes, increases soil erosion, higher surface temperatures and intensity of dry-seasons^2^, creates food insecurity^3,4^, as well as patterns and pace of species extinction^5,6^. Tropical forests as a key-category of land ecosystems include some of the world’s most diverse habitat types, while simultaneously being faced with the highest levels of biodiversity loss.

Deforestation (in addition to the burning of fossil fuel) is a large contributor to the increase of greenhouse gas concentrations in the atmosphere to the highest level of the last 20 Myr^7^. During this century, expected widening of the tropical climatic belt could promote distribution shifts in tropical plant communities (towards higher latitude and elevation), altering patterns of local/regional endemism and increasing rates of extinction (e.g. temperate taxa on tropical mountains)^8,9^. It has been suggested the tropics already expanded in the last few decades^10–14^.

Tropical lowlands in South East Asia experience some of the highest levels of deforestation rates globally^15^, followed by the establishment of monotypic plantation forest (or land is left fallow)^16^. In lowland tropical Asia, Dipterocarpaceae are a dominant ecological and structural component covering vast areas and economically, they are the most important and valuable source of timber products. Studies on the effects of selective logging (with removal of approximately one third of all trees) on vegetation in Kalimantan showed that harvesting removed 62% of dipterocarp basal area^17^. This high proportion stresses the significant physiognomic and ecological role of Dipterocarpaceae and understanding both evolution and the distribution of genomic diversity in this family. Despite their important ecological and economical values, we know comparatively little about Dipterocarpaceae genomics, its geographic distribution and their ability to adapt to changing environmental conditions. It is vital to better understand the evolution and ecology of subtropical taxa first, as these can be expected to respond quickly to any major changes in poleward expansion of tropical zones, leading to fundamental changes in ecosystems, their composition and functioning^18,19^.

Dipterocarpoideae (i.e. Asian Dipterocarps) are spread throughout the Indo-Malayan realm (i.e. most of the South and Southeast Asia and the southern parts of East Asia), as well as in the Indian subcontinent. Indeed, insights on the evolution of Dipterocarpaceae and their historical distribution have changed dramatically since Udvardy^20,21^. The northern limit for the family in the Indo-Malayan realm is located in subtropical southern China. Therefore, southern China represents a large “colonization front” for tropical tree species, and studies of species found in this area are particularly interesting for monitoring habitat ecology, community composition and adaptation to changing climatic conditions.

In Asia, Dipterocarpaceae are facing both climatic and anthropogenic challenges to their distribution, ecology and persistence (patterns of phenology in widespread species (*e*.*g*. *D*. *turbinatus*, occurring in Indo-Burma, Indonesia and the Philippines), distribution as a result of shifts in climatic conditions^22,23^, genetic diversity of species^24–26^).

**Figure 1:**
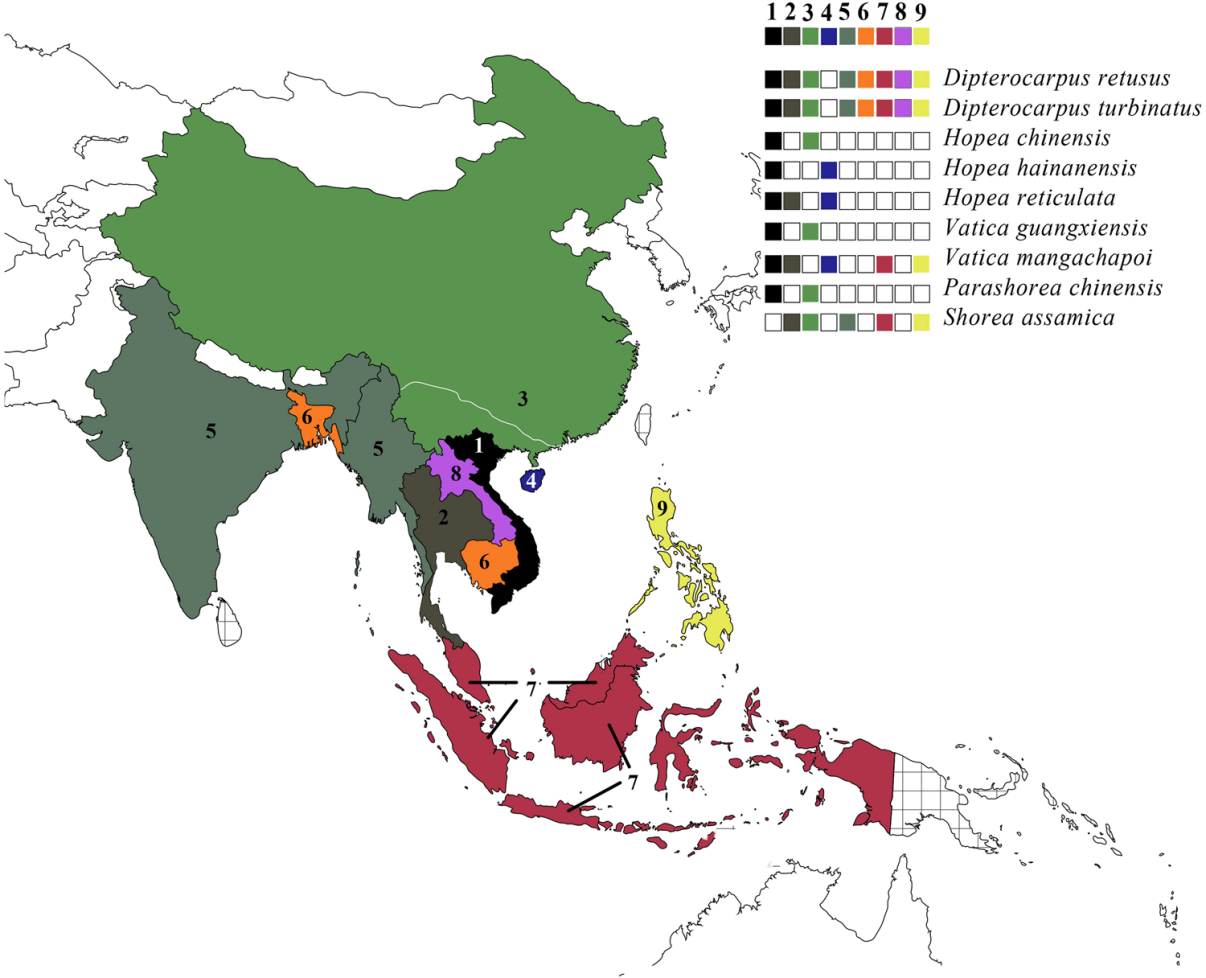
Dipterocarpaceae distribution in the northern SE Asian and China :1- Viet Nam, 2- Thailand, 3- mainland China (the distribution range for Dipterocarpaceae species in China is below white line), 4- Hainan, 5- India and Myanmar, 6- Bangladesh and Cambodia, 7- Malaysia and Indonesia, 8- Laos, 9- The Philippines; distribution of species represented by different colours; countries grouped together represent the places where species occur at both places, areas with mesh patterns represent all places where Dipterocarpaceae are occurring, but not part of this study (Distribution data collected from Flora of China^72^, Tropicos^73^, GBIF^74^, asianplant.net^75^, Kew’s Plants of the World Online^76^, Kew’s World Checklist of Selected Plant Families^77^).

Herein, we aim to provide the first assessment of the genomic diversity in Dipterocarpaceae species found at the tropics-subtropics boundary by (1) comparing genomic diversity of species found at the northern margin of the Dipterocarpaceae distribution range, namely the Southern Chinese provinces; (2) analyzing how the nucleotide variability is distributed in the plastome and the Nuclear Ribosomal Cistron (NRC); (3) comparing both the intrageneric and intergeneric nucleotide diversity, as China has high generic but relatively poor species diversity (Fig. 1).

## Materials and Methodology

The evergreen tropics (23.5°N to 23.5°S)^27^ is a zone with a non-arid, frost-free climate (only a wet and a dry season)^28^, while the subtropics are more loosely defined in their extent and location. Many plant taxa have their center of diversity in tropical regions, also have close relatives in adjacent subtropical zones^29^. Subtropical climates are characterized by warm to hot summers, and infrequent frost during cool winter^28^. Climate, geology, geography and time have shaped distinctive floristic compositions and species diversity that can be used to evaluate the dynamics of these forest communities^30^ positioned on the border of the subtropical and tropical zones. Indo-Malayan Dipterocarpaceae species are spread over tropical and subtropical region. Here we focus on the northernmost fringe of the Dipterocarpaceae distribution (northern SE Asia and China), and the species contained within this region (Fig. 1).

Individuals were collected in China (Hainan and Yunnan), both in the wild and in botanical gardens. Voucher materials were deposited in the Biodiversity Genomics Team herbarium (BGT), Nanning, China (see accession list Supplementary Table S1).

In this study we used six species belonging to the tribe Dipterocarpeae (*Dipterocarpus turbinatus* C.F. Gaertn., *D*. *retusus* Blume, *D*. *alatus* Roxb., *Vatica mangachapoi* Blanco, *V*. *guangxiensis* S.L. Mo and *V*. *rassak* (Korth.) Blume), and five species belonging to the tribe Shoreae (*Hopea chinensis* (Merr.) Hand.-Mazz., *H*. *hainanensis* Merr. & Chun, *H*. *reticulata* Tardieu, *Shorea assamica* Dyer and *Parashorea chinensis* Wang Hsie). Nine species of both tribes occur in China, and represent five genera in the subfamily Dipterocarpoideae, which contain almost half of all genera found in Asia. Due to continued uncertainty regarding the relationships of Dipterocarpaceae with other families in Malvales, we added eight outgroups spread throughout the order: (*Aquilaria sinensis* (Lour.) Gilg, *Theobroma cacao* L., *Talipariti hamabo* (Siebold & Zucc.) Fryxell, *Hibiscus syriacus* L., *Gossypium barbadense* L., *Tilia amurensis* Kom., *Abelmoschus esculentus* (L.) Moench, *Daphne kiusiana* Miq.) and *Arabidopsis thaliana* (L.) Heynh. We also added two additional species of Dipterocarpaceae (*Dipterocarpus alatus* and *Vatica rassak*) with a more southern distribution (SE Asia; Indonesia, Philippines and Papua New Guinea, respectively).

### DNA extraction and sequencing

Total genomic DNA was purified from 0.1 g frozen fresh leaves with the Plant Genomic DNA Kit (Tiangen Biotech Co., Ltd), following manufacturer instructions. After purification a 350-bp paired-end library was constructed using the NEBNext Ultra II DNA Library Prep Kit (Ipswich, Massachusetts, USA). Raw data were sequenced with the Illumina HiSeq2500 platform (San Diego, California, USA), with a paired-end read length of 2 × 150 bp. Libraries construction and sequencing were performed by Novogene (Beijing, China).

### Dataset construction

To reconstruct high-quality plastomes, we used a genome skimming approach (i.e. bioinformatic sorting of highly repetitive genomic sequences, *e*.*g*. the plastome, the mitochondrial genome and the NRC) combined with both *de-novo* and reference-guided assembly. Because of the absence of complex structural rearrangements in the chloroplast genome, plastomes are more suitable for phylogenomic analyses than mitochondrial genomes. To investigate the presence of phylogenomic incongruence between the maternally inherited chloroplast genome and the paternally inherited nuclear genome, we also reconstructed the NRC.

### Plastomes assembly

Draft chloroplasts were reconstructed with ORG.Asm v0.2.05^31^, using default settings. The raw reads and the resulting circular contigs were both imported in Geneious R10 v.10.0.5 (http://www.geneious.com)^32^. Raw reads were trimmed, removing bases from 5′ and 3′ –ends until all were with quality Q < 20 (i.e., with sequencing error rate lower than 1%). As some of the ORG.Asm assemblies resulted in several shorter linear contigs, these were extended by an iterative mapping approach in Geneious R10, until matching ends were found and a circular plastome could be constructed. The removal of assembly errors was done manually during the process of assembly and alignment.

To assess the assembly quality, reads were mapped against the curated ORG.Asm assembly or the consensus sequence from the iterative mapping, using a reference-guided method. Reads with less than 10 low quality bases and/or ambiguities were mapped using the Geneious R10 mapper. Using the ORG.Asm circular contig as a reference, the algorithm iteratively maps the reads against the reference, starting with the most conserved regions. These first contigs are then used as a “pseudo-reference” and refined or extended with the partially overlapping reads newly mapped. 1,000 iterations were performed with gaps allowed (up to 15% of the reads length), a word length of 14 bp and an index word length of 12 bp. The maximum mismatch per read and maximum ambiguities were set to 30% and 4, respectively. The “Accurately map reads with errors to repeat regions” option was checked, only reads assembled to the correct distance (i.e. ≈350–500 bp) were considered by the Geneious algorithm, and this information was used for scaffolding.

Positions under 5x coverage were masked (Ns) for the generation of a consensus sequence, despite we individually checked these positions to verify the base calling accuracy and their identity to the reference. These positions were accounted for the length calculation. The inverted repeat (IR) borders were carefully checked by eye for each species, no evidence for any structural change of these IRs borders were found, as the mapping depth and base calling were without ambiguity. We determined annotations for each newly sequenced species using cpGAVAS^33^, followed by manual adjustments. Sequences were aligned using the Geneious MAFFT v7^34^ implementation in Geneious R10 with default settings.

### Assembly of extended nuclear ribosomal cistron regions

Available nITS data on Genbank for the species in our study was limited to three species (*Vatica mangachapoi*, *Hopea hainanensis* and *P*. *chinensis*). Using *P*. *chinensis* (GenBank KR532475), we generated the extended NRC of all Dipterocarpaceae species in our study using an iterative mapping approach (i.e. reference-guided assembly), with medium-low sensitivity, and 1000 iterations. The NRC sequences were then annotated using *Theobroma grandiflorum* as a reference (GenBank JQ228378) in Geneious. Sequences were aligned using the Geneious R10 alignment with free end gaps. The complete plastomes and nuclear ribosomal cistrons will be submitted to DRYAD upon acceptance.

### Intergeneric comparisons, interspecific comparison and phylogenomic reconstruction

A maximum likelihood (ML) tree was built using RAxML-NG v0.8.1^35^. For evaluation of node supports we used standard bootstrap analyses with 1000 replicates^35^. In addition to plastomes ML tree, separate phylogenies for the Large Single Copy (LSC) regions, Small Single Copy (SSC) regions, and IR were built using the same parameters. We used ModelTest-NG v0.1.5^36^ for choosing the model for our datasets (GTR + G4 for plastomes, NRC and LSC; GTR + I + G4 for total evidence of plastomes and NRC, TVM + G4 for SSC; TVM + I + G4 for IR) to find the best ML tree. ML trees were edited with the program FigTree v1.4.3^37^ [http://treebioedacuk/software/figtree/]. We also conducted phylogenomic analyses on a combined plastome-NRC dataset using MrBayes v3.2^38^. Metropolis-Coupled Markov Chain Monte Carlo (*MC*^3^) sampling was performed with four chains running for 20 * 10^6^ generations, sampling every 1000^th^ generation and discarding the first 50% of sampled trees as burnin. We used the percentage of pairwise identity and the pattern of identities and regions of mismatch between two sequences to calculate pairwise distance. Repeated sequences for each species (forward, palindrome, reverse and complement sequences) were identified using REPuter^39^ as previously described (e.g.^40,41^), with 30 bp and greater than 90% sequence identity. Simple sequence repeats (SSRs) were found using MISA^42^ with minimum number repeats of 10, 5, 4, 3, 3 and 3 for mono-, di-, tri-, tetra-, penta- and hexa- nucleotide, respectively. To identify regions with substantial variability, the complete plastomes of eleven Dipterocarpaceae species, eight species of Malvales and *Arabidopsis thaliana* were compared using mVISTA^43,44^, using *Dipterocarpus turbinatus* as a baseline for comparison of all other plastomes.

## Results

We reconstructed plastomes and NRC sequences for nine Dipterocarpaceae species occurring in China and two additional species of Dipterocarpaceae (see accession list Supplementary Table S1).

### Plastomes sizes and features

The plastomes lengths of our selected species ranged from 151,033 bps (*Vatica guangxiensis*) to 156,706 bps (*Dipterocarpus turbinatus*) (Supplementary Tables S2, S3). All individuals exhibited the typical organization of the chloroplast, with LSC, SSC regions and two IR copies of approximately 85 kbps, 20 kbps and 24 kbps, respectively. The overall GC content of all analyzed species was 35.2%, 31.9% and 43.1% in LSC, SSC and IR regions, respectively. In total, 125 genes were annotated, including around 88 protein coding genes, 31 tRNA genes and 8 rRNA genes.

### NRC dataset characteristics

The NRC lengths of reconstructed sequences for the analyzed Dipterocarpaceae species ranged from 5,787 bps (*Dipterocarpus turbinatus*) to 5,830 (*Hopea hainanensis*) (Supplementary Table S4). NRC mapping depths were between 892X and 4,881× (*Vatica mangachapoi* and *Dipterocarpus turbinatus*, respectively) (Supplementary Table S4). 18S and 26S ribosomal RNA genes were highly conserved, whereas the 5.8S RNA gene showed higher level of variations. The most variable regions were both internal transcribed spacers, ITS1 (identical sites: 321; 85.1 pairwise % identity) and ITS2 (identical sites: 156; 88.5 pairwise % identity).

### Repeat and SSR analyses

**Figure 2:**
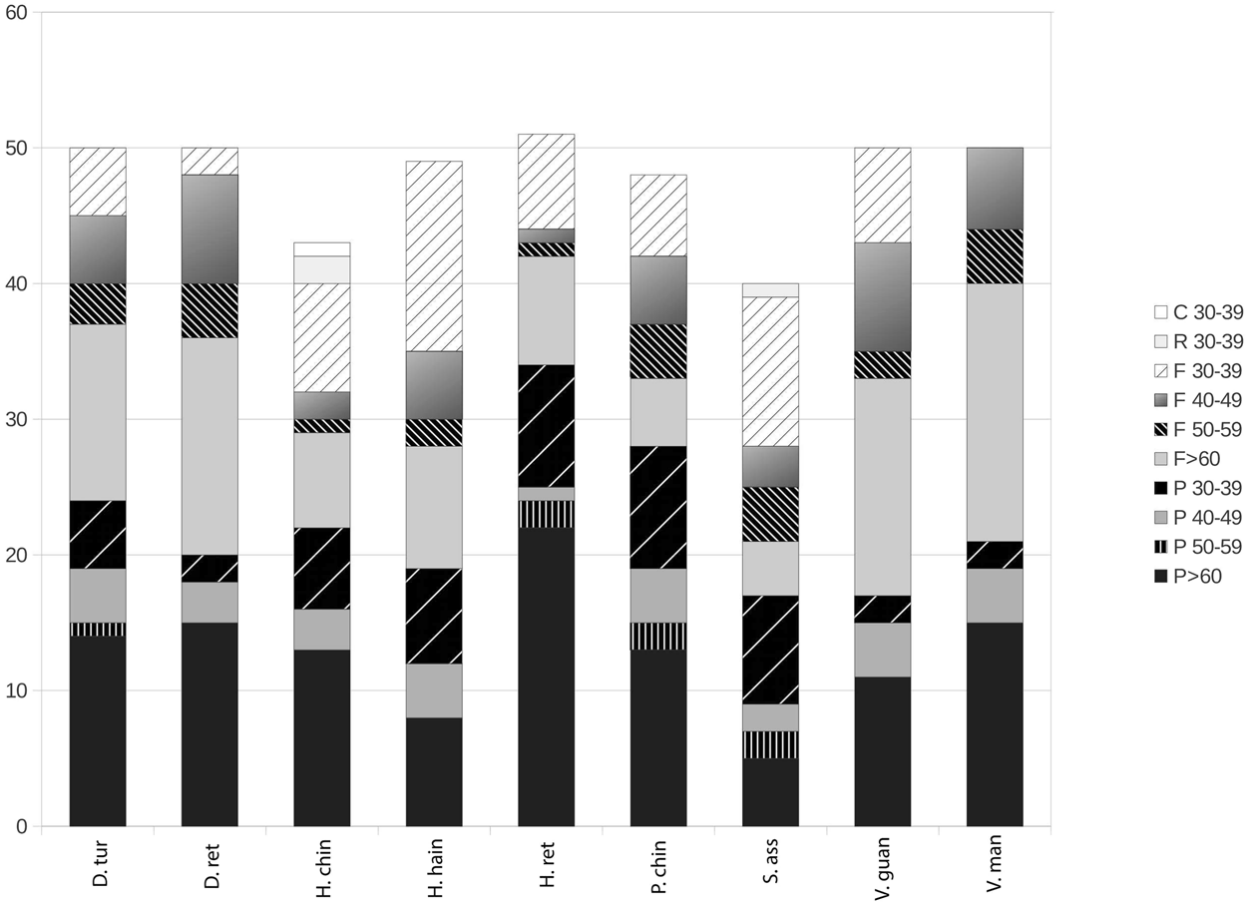
Repeat sequences in nine plastomes of Dipterocarpaceae. Repeat sequences with length ≥30 bp and sequence identity ≥90% in the plastomes were identified by REPuter. C, R, F and P indicate repeat type C (complement), R (reverse), F (forward) and P (palindrome), respectively. Repeats with different lengths are indicated by different patterns. D.ret: *Dipterocarpus turbinatus*; D.tur: *Dipterocarpus retusus*; H.chin: *Hopea chinensis*; H.hain: *Hopea hainanensis*; H.ret: *Hopea reticulata*; P.chin: *Parashorea chinensis*; S.ass: *Shorea assamica*; V.guan: *Vatica guangxiensis*; V.man: *Vatica mangachapoi*.

Using REPuter^39^, 50 repeats were found in *Dipterocarpus turbinatus*, *D*. *retusus*, *Hopea reticulata*, *Vatica guangxiensis* and *V*. *mangachapoi*, 49 in *H*. *hainanensis*, 48 in *Parashorea chinensis*, 43 in *H*. *chinensis*, 40 in *Shorea assamica* (see Fig. 2). Reverse (R) and complement (C) repeats were found only in *H*. *chinensis* and in *S*. *assamica*, represented by the lowest number of repeat sequences (1 complement and 2 reverse repeats with length of 30–39 bps in *H*. *chinensis* and 1 reverse repeat with length of 30–39 bps in *S*. *assamica*). In all species repeats were mainly distributed in the intergenic spacer regions between CDS and transfer RNA genes (Supplementary Tables S5 and S5.1). Interspecific comparison and analyses in the Chinese Dipterocarpaceae showed that *S*. *assamica* had the fewest number of repeats^40^. Forward and palindrome repeat sequences longer than 60 bp in the plastomes, followed by forward repeat sequences with a length of 30–39 bps (Fig. 2). Our results showed no clear pattern or structure according to the taxonomy.

**Figure 3:**
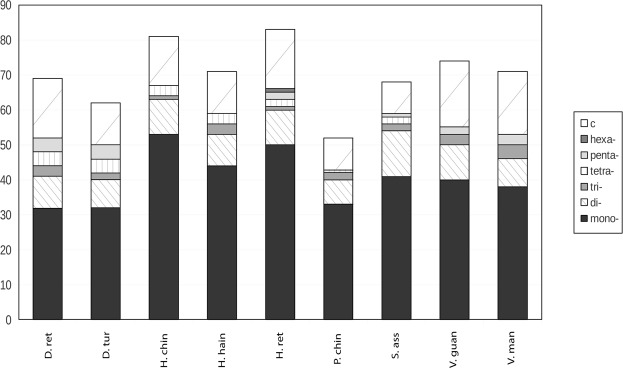
Number of simple sequence repeats in nine plastomes of Dipterocarpaceae. (mono-: mononucleotide SSRs; di-: dinucleotide SSRs; tri-: trinucleotide SSRs; tetra-: tetranucleotide SSRs; penta-: pentanucleotide SSRs; hexa-: hexanucleotide SSRs; c-: c-nucleotide SSRs. D.ret: *Dipterocarpus turbinatus*; D.tur: *Dipterocarpus retusus*; H.chin: *Hopea chinensis*; H.hain: *Hopea hainanensis*; H.ret: *Hopea reticulata*; P.chin: *Parashorea chinensis*; S.ass: *Shorea assamica*; V.guan: *Vatica guangxiensis*; V.man: *Vatica mangachapoi*.

Microsatellite regions in assembled plastomes showed differences in their numbers, with congeneric species showing dissimilarities in both numbers and spatial patterns of SSRs occurrence (Fig. 3). The highest number of SSRs was in the plastome of *Hopea reticulata*, and the lowest in *Parashorea chinensis*. Mononucleotide (approximately 50%) in all species. In the IGS region, 86.53% of all SSRs (mono-, di-, tri-, tetra-, penta-, hexa-, c- nucleotide) were found, and remain 13.47% in CDS genes (Supplementary Tables S6 and S6.1). All analyzed species lacked some SSRs patterns (G mononucleotides and GA dinucleotides), with C mononucleotide found only in *H*. *reticulata*.

### Dipterocarpaceae phylogenomic reconstruction and comparison

**Figure 4:**
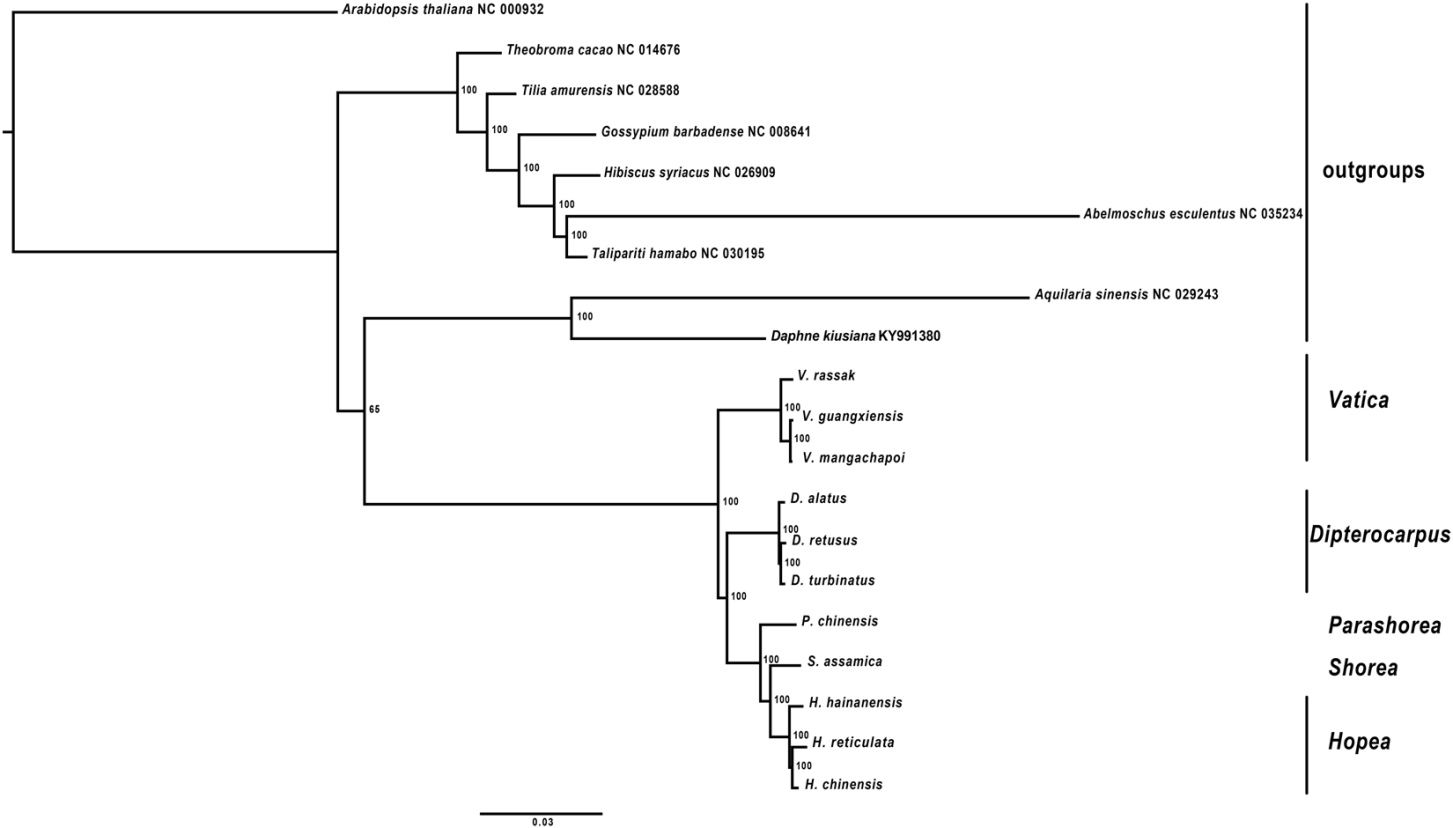
Maximum Likelihood phylogenomic tree of Chinese Dipterocarpaceae plastomes sequences, in addition to *Dipterocarpus alatus* and *Vatica rassak* and outgroups sequences, retrieved from GenBank. Bootstrap branch support shown at nodes.

Nodes in the ML tree based on plastomes were highly supported (bootstrap support = 100) (Fig. 4). *Vatica* diverged in the basal position with *Dipterocarpus- Parashorea- Shorea- Hopea* clade. Intrageneric branches in nearly all Dipterocarpaceae are very short, highlighting the low levels of genomic diversity among species. *Dipterocarpus* and *Hopea* formed distinct clades, interspersed with *Parashorea chinensis* and *Shorea assamica* (Fig. 4). Pairwise identity values underscore the low levels of variation (*Vatica guangxiensis* vs. *V*. *mangachapoi* share 99.4 pairwise % identity (126,811 identical sites), while *Dipterocarpus alatus* is different from Chinese species *D*. *turbinatus* and *D*. *retusus* (98.7 pairwise % identity; 128,274 identical sites). *Hopea hainanensis* diverged in a basal position in the genus relative to *H*. *reticulata* and *H*. *chinensis* (97.1 pairwise % identity; 124,614 identical sites). Substitutions and indels were spread throughout the aligned sequences of *V*. *rassak* and *D*. *retusus* (92.2 pairwise % identity, 121,685 identical sites).

Phylogenies of the LSC, SSC regions, and IR, with some variation in the level of diversity confirmed the same evolutionary history as in the plastomes (see Supplementary Figs S1–S3).

*Vatica rassak*, widespread in Indonesia (Buru), Papua New Guinea and the Philippines, occupies a basal position compared to more northerly distributed *V*. *mangachapoi* and *V*. *guangxiensis*. *Dipterocarpus turbinatus* and *D*. *retusus* can be found from SE Asia to Myanmar-India, with the distribution range of *D*. *retusus* extending to Malaysia, Java and the Philippines. *D*. *alatus* with narrow, southern distribution from Thailand to Cambodia, is in a basal position to the Chinese Dipterocarps. *Shorea assamica* is widespread from India to the Philippines, whereas all analysed *Hopea* species, as well as *Parashorea chinensis* have a restricted distribution South China, North Viet Nam and Thailand.

### Comparative interspecific NRC and plastome genomic analyses

**Figure 5:**
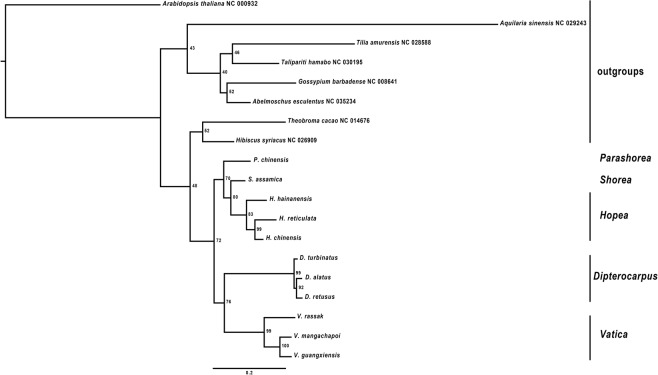
Maximum Likelihood phylogenomic tree of Chinese Dipterocarpaceae NRC sequences, with *Dipterocarpus alatus* and *Vatica rassak*. Outgroups sequences were retrieved from GenBank. Bootstrap branch support shown at nodes.

In the NRC ML tree, *Hopea* formed a robustly supported clade, distinct to the *Dipterocarpus* and *Vatica* clade (moderate to highly bootstrap support: 70–100, see Fig. 5). Intrageneric branching order in *Dipterocarpus* was different from the plastomes ML tree. In the NRC tree, *D*. *turbinatus* was highly associated (bootstrap support = 99) in the basal position with the *D*. *alatus- D*. *retusus* clade. There were no ingroup differences of analyzed species belonging to the genus *Hopea* and *Vatica* between the NRC and plastomes trees (Fig. 5).

### Total evidence ML phylogenomic tree

**Figure 6:**
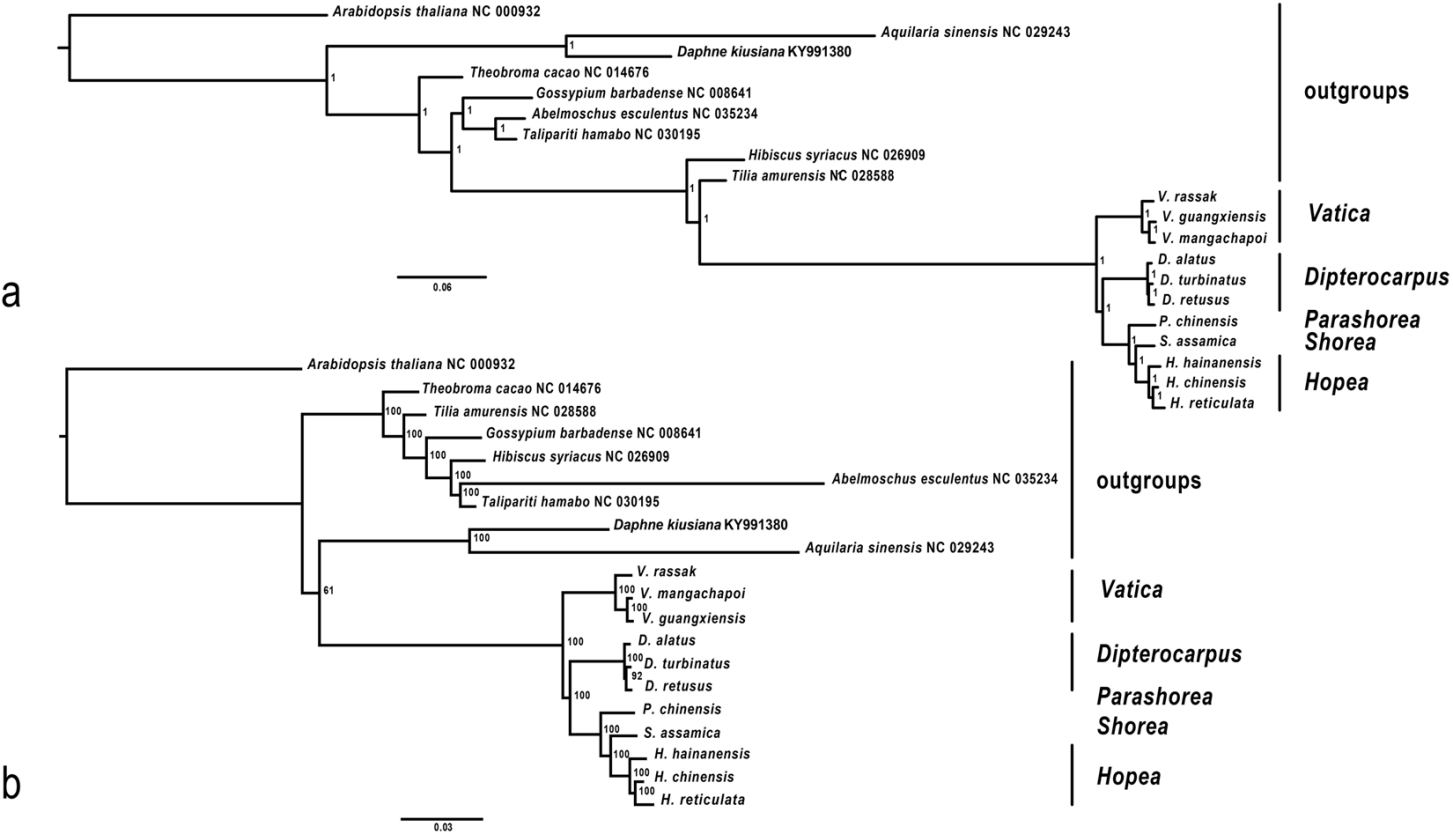
Total evidence (plastomes + NRC) of the complete dataset of Chinese Dipterocarpaceae, *Dipterocarpus alatus* and *Vatica rassak*, and outgroups retrieved from GenBank. (**a**) MrBayes phylogenomic tree. (**b**) Maximum Likelihood phylogenomic tree (bootstrap support values shown at nodes).

Bootstrap supports for the ML tree of the combined complete plastomes and NRC were high (≥92) (Fig. 6b). The branch leading to the genus *Vatica* diverged from a clade containing *Dipterocarpus*, *Parashorea chinensis*, *Shorea assamica* and *Hopea*, in agreement with the plastomes ML tree. In addition, the genus *Dipterocarpus* diverged from a clade containing *P*. *chinensis*, *S*. *assamica* and the genus *Hopea*. *D*. *alatus* was associated in the basal position with the *D*. *turbinatus*- *D*. *retusus* clade, like in the plastomes ML tree, as well as *Hopea hainanensis* in the genus *Hopea*. The four trees retrieved the same placement for *Vatica* (Fig. 6b).

### Total evidence MrBayes phylogenomic tree

The tree generated with MrBayes v3.2^38^ using complete plastomes and NRC sequences showed highly supported nodes throughout (PP:1). It displayed the same topology like the ML tree (Fig. 6a).

### Comparative plastome analyses

**Figure 7:**
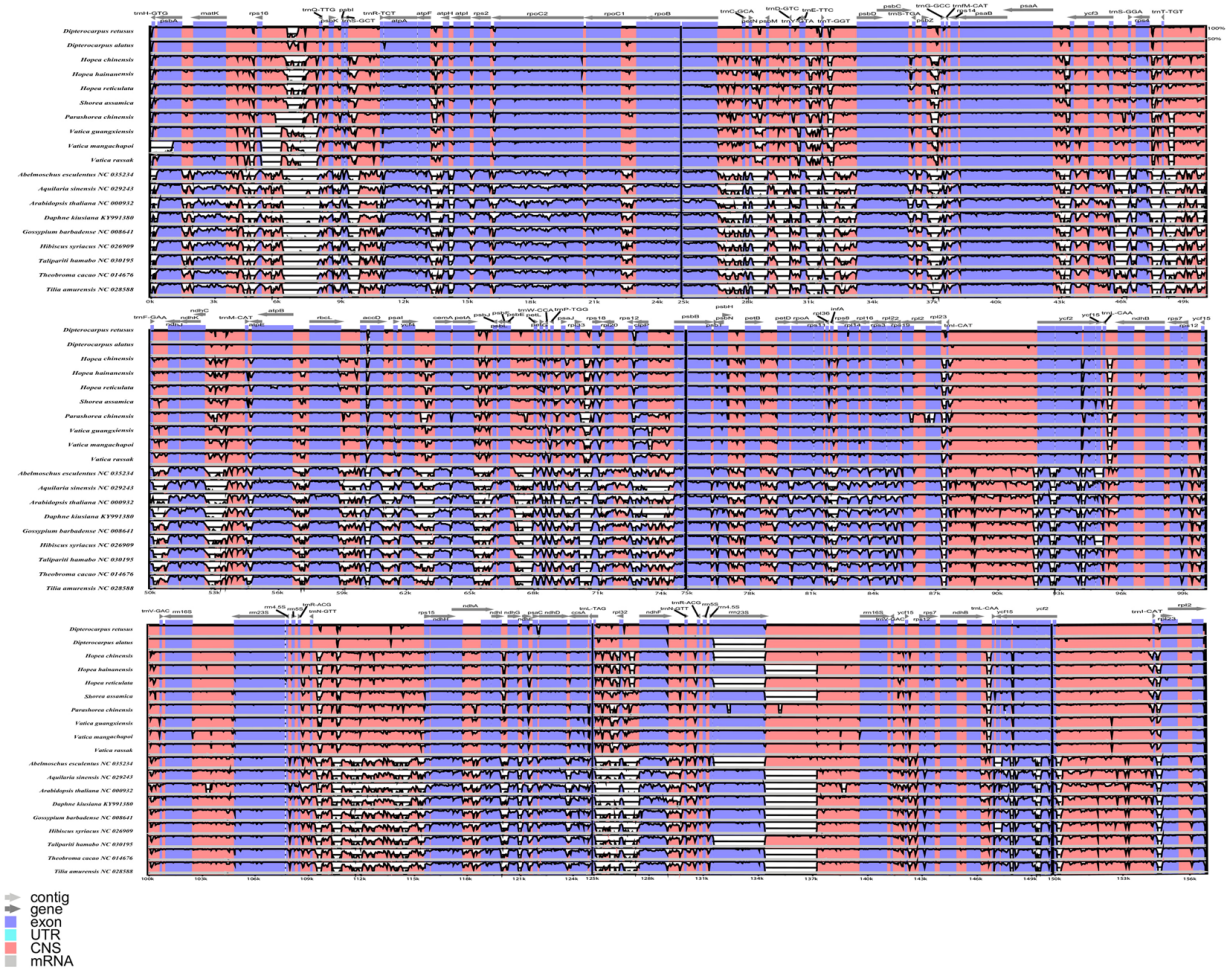
mVISTA alignment and percent identity plot of plastomes of Dipterocarpaceae and outgroups. Similarity is expressed relative to *Dipterocarpus turbinatus*, used as a reference for annotations. Arrows represent the transcriptional direction. Vertical scale indicates the percentage of identity ranging from 50% to 100%. Coding regions are in blue while non- coding are in pink.

Interspecific analysis using mVISTA^43,44^ of eleven Dipterocarpaceae species, eight species of Malvales, plus *Arabidopsis thaliana*, showed the plastomes of Chinese Dipterocarpaceae species were highly conserved in their structures (Fig. 7). Among coding regions, the most conserved coding regions were *rpl23*, *rrn16S*, *ndhB*, *rps7*, *psbD*, *psbC*, whereas *atpF*, *atpA*, *trnR-TCT*, *ndhK*, *ndhJ*, *rpoC2*, *rps4*, *ccsA* and *ycf2* coding regions were the most variable. The highest level of variations was detected in non-coding regions that could therefore be used for phylogenetic analyses. The most variable non-coding regions were the *rps16-trnQ-TTG*, *rpl33-rps18*, *trnL-ndhB*, *trnN-rps15*, *trnL-rpl32*, *rpl32-ndhF* spacers, and between the *16S* and *23S* ribosomal subunits. The most conserved non-coding regions were the *ndhB-rps7* and, *trnI-ycf2* spacers (Fig. 7). Notably, several major deletions (50%) in both outgroups and ingroups occur in *23S* ribosomal subunits.

## Discussion

The number of publicly available plastomes, despite growing rapidly with the technical development, remains very limited in Dipterocarpaceae^45,46^. Our previous study^45^ highlighted the relationships of major lineages in Malvales were not congruent with those previously published elsewhere. Here, we provide genomics resources in an attempt to place our dataset in a broader taxonomic framework and further clarify those relationships. In this study, we generated 11 new complete plastomes of Dipterocarpaceae and compared them in combination with a set of other taxa spread throughout Malvales and *Arabidopsis thaliana*. Dipterocarpaceae in China are distributed throughout five different genera. Comparative analyses confirmed different levels of variation in repeated sequences in plastomes, but similar SSRs diversity and characteristics.

Microsatellites [(SSRs) or short tandem repeats (STR)] are a commonly used marker system in plant genetics and breeding^42^. Chloroplastic microsatellites were widely used in population genetics of Dipterocarpaceae to assess genetic variation and population spatial structure at lower spatial and temporal scale^47–49^, and the genetic diversity and gene flow with closely related species^50^. Our analyses of repeated regions, microsatellites, and the comparative plastomes analyses showed that a higher portion of the number and characteristics variations were found between species than between genera. This suggest that microsatellites loci can be more efficient in delineating closely related species than either distant species or genera.

In Malvales, phylogenetic relationships among Cistaceae, Dipterocarpaceae and Sarcolaenaceae remain unclear^51^. Using either single-locus approached [*rbcL*^52,53^], or multi-loci methods [*rbcL*, *trnK-matK-trnK* and *trnT-trnL-trnF* plastid regions^54^] previous studies still have not certainly resolved their evolutionary placement. Using both chloroplast (*atpB*, *matK*, *ndhF*, and *rbcL*) and mitochondrial (*matR*) loci to reconstruct a phylogenomic tree of Chinese vascular plants^55^, Chen *et al*. retrieved a paraphyletic Dipterocarpaceae family. In addition, previous studies highlighted the unresolved phylogenetic placement of Dipterocarpaceae^50,54,56^, as well as its uncertain origin (despite the monophyly of the family is supported by a common ectomycorrhizal ancestor^57,58^). We found low levels of genomic variation in the family, although our sampling was not designed to test monophyly of the family.

Short terminal branches in our analyses and similar patterns of variations in plastomes for all Chinese species highlight the low levels of genomic variation in the family. These short internal branches suggest a recent rapid diversification of Dipterocarpaceae at their Northern distribution range and elsewhere, as found in other taxonomic groups highlighting recent diversifications during the Oligocene and the Miocene (e.g.^59^). However, our sampling - a small fraction of all species in the family - cannot be used for dating, as it would be strongly sensitive to any error in substitution rate estimations. Moreover, unreliable and rare fossils of the family on nodes that are likely evolutionary distant that the actual nodes a fossil should calibrate, could result in a high level of uncertainty. Nonetheless, our genomic resources could be the base for further more detailed and accurate calibration studies.

In Dipterocarpaceae, relationships between *Hopea*, *Shorea* and *Parashorea* remain unclear^60–63^. In our study *Shorea* is close to *Parashorea* than to *Hopea* clade, opening questions for still uncertain phylogenetic relationships among the two biggest genera in the family. Phylogenetic placement of two genera *Dipterocarpus* and *Vatica* is still uncertain^54,61,63^. The incongruent NRC and plastome datasets highlighted complex and different ways of evolution of these genera. Even though hybridization among *Hopea* and *Shorea* could be one of the reasons for the incongruence between plastome and NRC data, further studies are needed to conclude.

By nature, standardized markers widely used across large taxonomic ranges show low level of variation^64,65^. Although useful for studies at higher taxonomic levels, this severely limits their power to delineate phylogenetic relationships in large, complex, evolutionary young groups like Dipterocarpaceae^56^. Using a genome-based approach combining plastomes with NRC sequences, we obtained a dataset of more than 160,000 bp that provides a robustly supported backbone at the family and the genus level, but also the relationships among included species.

NRC sequence, (i.e. tandemly repeated transcription units of the nuclear ribosomal DNA, or nrDNA) consist of an intergenic spacer (IGS), ITS 1 and 2 (ITS1, ITS2), the 5.8S rDNA gene, the small- (SSU- 18S) and the large-subunit (LSU- 5.8S and 28S) rDNA gene. The first part of the IGS (commonly referred as ETS - External Transcribed Spacer) and the two ITS regions have higher level of substitution rates due to their relaxed functional constraint and rapid evolution^66^. rRNA genes are usually more conserved, because their transcripts are directly involved in the formation of ribosomes^66–68^, and thus highly constrained. Our interspecific NRC analyses showed similar length of NRC. Only 5.8S rDNA gene had higher level of variability than expected, according to its conserved structure, likely due to the lack of a set of properly annotated sequences in databases used for our annotations transfer. Indeed, the exact boundaries of the rDNA genes were determined using the relatively distant, *Theobroma grandiflorum* as a reference.

Finally, because Dipterocarpaceae are the economical cornerstone of tropical forestry in Asia, generating ecological, genomic and morphological resources is an essential step towards conservation of genetic resources and long-term sustainable use.

## Conclusions

Here, we assembled a dataset comprising nearly all Dipterocarpaceae species occurring at the Northernmost margin of the family and several core-Asian species (both in the family and in Malvales *s*.*l*.). Comparison of genomic diversity at different taxonomic levels (intergeneric and intrageneric) showed low level of genomic diversity among analysed range-edge species, and the incongruence between plastome and NRC data.

Our study provide genomics resources for further detailed assessment of these species characteristics (e.g. demography, population structure, admixture history^69^), and a base to understand how edge-species distribution respond to changing environment in terms of their abundance, range of distribution and extinction, as highly vulnerable groups^49,70,71^. It could therefore be used for further comparative analyses between Chinese Dipterocarpaceae occurring on the edge, and SE Asia Dipterocarpaceae species in the core of their distribution, conservation and sustainable use of these crucial Asian forest resources, and the reconstruction of the evolutionary history of the whole Dipterocarpaceae family.

## Supplementary information

Supplementary Information

Dataset 1

Dataset 2
