# Supplementary material for: Exploring evolution and diversity of Chinese Dipterocarpaceae using next-generation sequencing
Source: Sci Rep. 2019 Aug 12;9:11639. doi: 10.1038/s41598-019-48240-y (PMC6690942; doi:10.1038/s41598-019-48240-y)
Supplement: Supplementary file 1 — Supplementary Information [file 41598_2019_48240_MOESM1_ESM.pdf]

Supplementary Information

Article in *Scientific Reports*

Exploring evolution and diversity of Chinese Dipterocarpaceae using next-generation sequencing

Tijana Cvetković[1]; Damien Daniel Hinsinger[13]; Joeri Sergej Strijk[123*]

[1] Biodiversity Genomics Team, Plant Ecophysiology & Evolution Group, Guangxi Key Laboratory of Forest Ecology and Conservation, College of Forestry, Daxuedonglu 100, Nanning, Guangxi, 530005, PR China, [2] State Key Laboratory for Conservation and Utilization of Subtropical Agro-bioresources, College of Forestry, Guangxi University, Nanning, Guangxi 530005, PR China, [3]Alliance for Conservation Tree Genomics, 530005, ChinaPha Tad Ke Botanical Garden, PO Box 959, 06000 Luang Prabang, Lao PDR

[*] **Corresponding author:** jsstrijk@hotmail.com

Supplementary Figures:

Supplementary Figure S1:

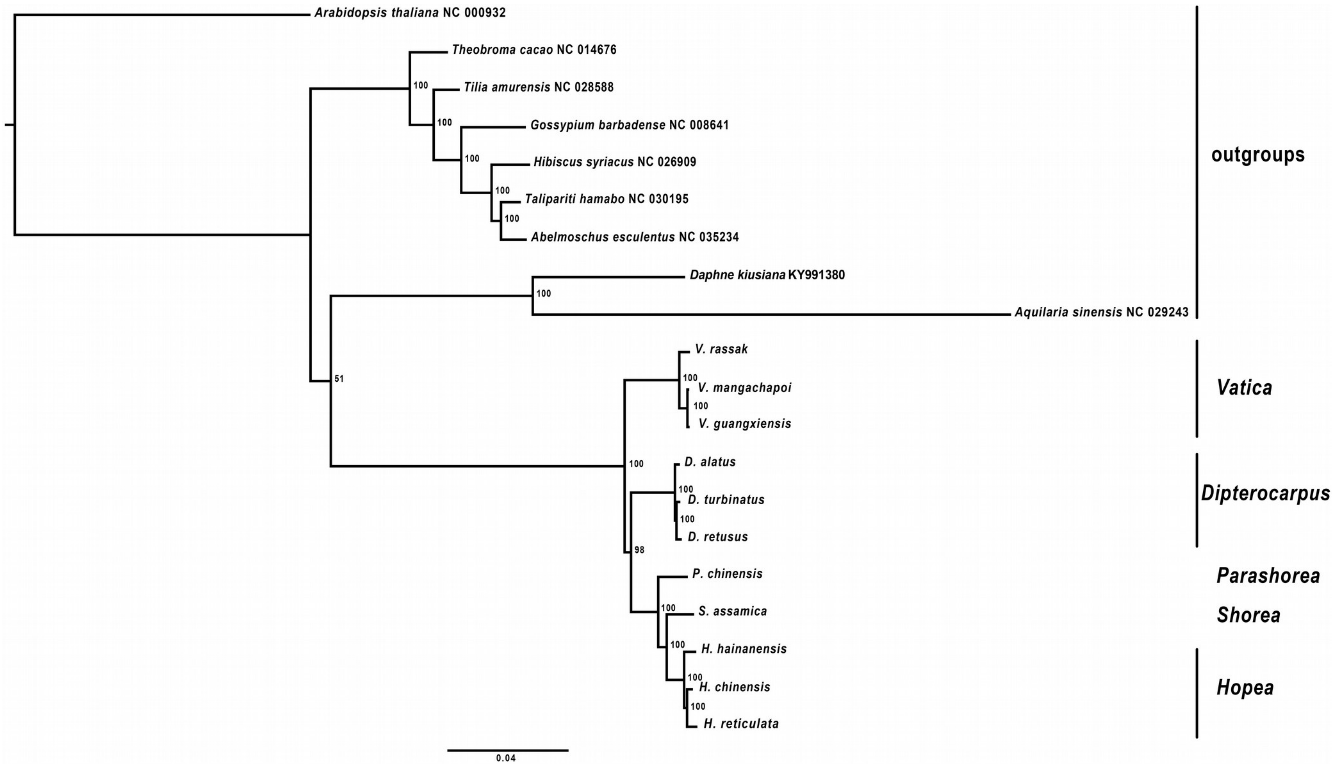

Supplementary Figure S1. Maximum Likelihood phylogenomic tree of Chinese Dipterocarpaceae LSC sequences, with *Dipterocarpus alatus* and *Vatica rassak*. Outgroups sequences were retrieved from GenBank. Bootstrap branch support shown at nodes.

Supplementary Figure S2:

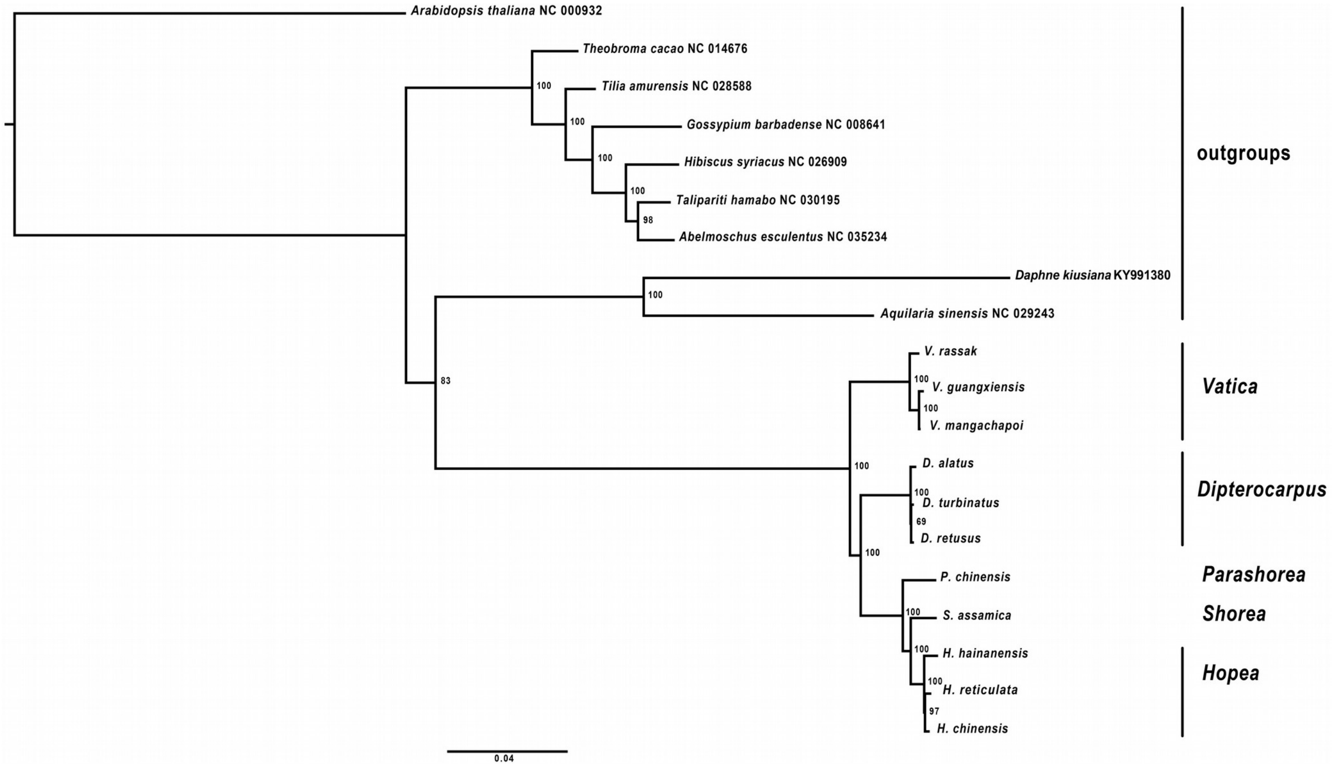

Supplementary Figure S2. Maximum Likelihood phylogenomic tree of Chinese Dipterocarpaceae SSC sequences, with *Dipterocarpus alatus* and *Vatica rassak*. Outgroups sequences were retrieved from GenBank. Bootstrap branch support shown at nodes.

Supplementary Figure S3:

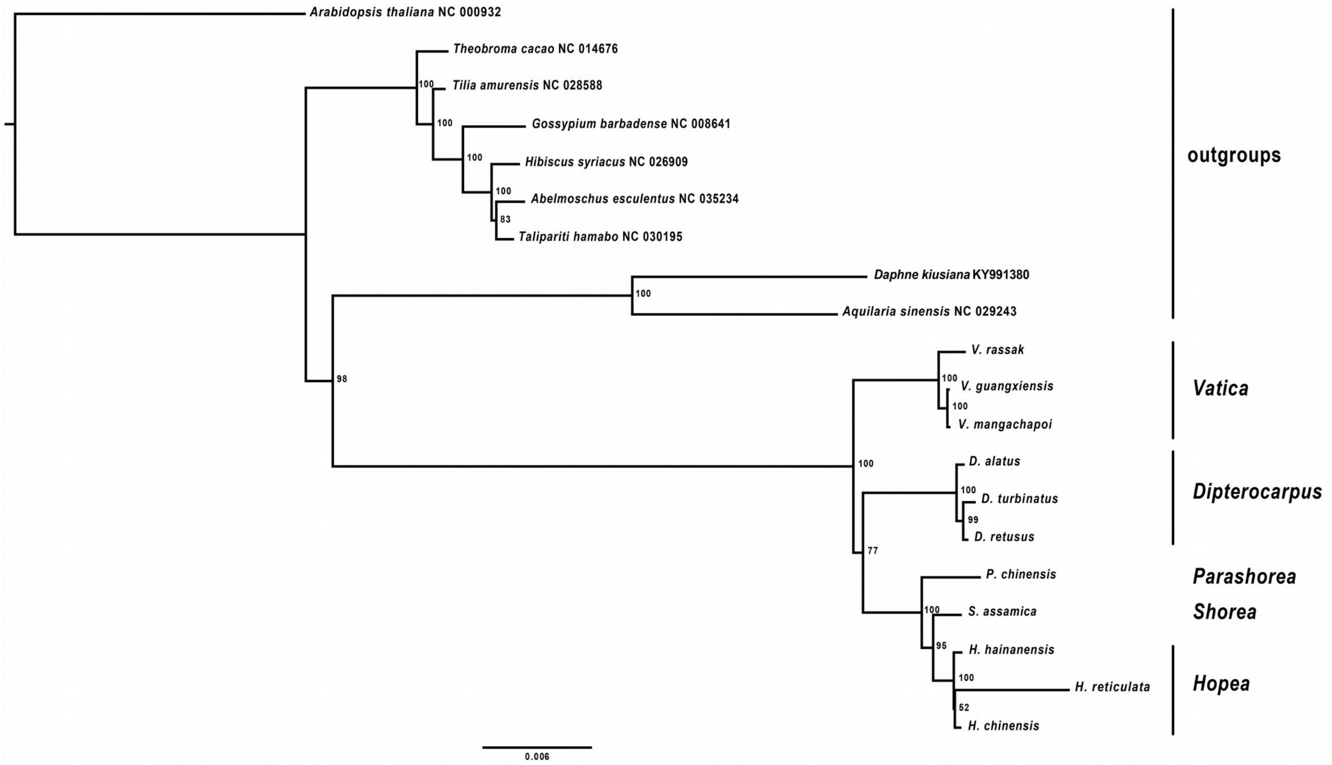

Supplementary Figure S3. Maximum Likelihood phylogenomic tree of Chinese Dipterocarpaceae IR sequences, with *Dipterocarpus alatus* and *Vatica rassak*. Outgroups sequences were retrieved from GenBank. Bootstrap branch support shown at nodes.

Supplementary Tables:

Supplementary Table S1:

Species name	Collecting locality	cpDNA DRYAD Accession No.	cpDNA GenBank Accession No.	ITS GenBank Accession No.	NRC DRYAD Accession No.	Voucher information & BGT herbarium
Dipterocarpus alatus Roxb.	China	X	-	-	X	BGT 1847
D. retusus Blume	Yunnan, China	X	-	-	X	Strijk 1610
D. turbinatus C.F.Gaertn.	Yunnan, China	X	-	-	X	Strijk 3740
Hopea chinensis (Merr.) Hand.-Mazz	Yunnan, China	X	-	-	X	Strijk 1613
H. hainanensis Merr. & Chun	Guangxi, China	X	-	-	X	Strijk 1563
H. reticulata Tardieu	Yunnan, China	X	-	-	X	Strijk 1597
Parashorea chinensis Wang Hsie	Yunnan, China	X	-	KR532475	X	Strijk 3594
Shorea assamica Dyer	China	X	-	-	X	Strijk 1602
Vatica guangxiensis S.L. Mo	Guangxi, China	X	-	-	X	Strijk 1561
V. mangachapoi Blanko	Yunnan, China	X	-	-	X	Strijk 1605
V. rassak (Korth.) Blume	Yunnan, China	X	-	-	X	Strijk 1591
Abelmoschus esculentus (L.)Moench	Austin, USA	-	NC035234	KP222461	-	-
Aquilaria sinensis (Lour.) Gilg	Hainan, China	-	NC029243	GQ891956	-	-
Arabidopsis thaliana L.	USA	-	NC000932	DQ528813	-	-
Daphne kiusiana Miq.	Korea	-	KY991380	-	-	-
Gossypium barbadense L.	Kyoto, Japan	-	NC008641	GU935141	-	-
Hibiscus syriacus L.	Korea	-	NC026909	KM117267	-	-
Talipariti hamabo Siebold & Zucc.	China	-	NC030195	KX984261	-	-
Theobroma cacao L.	Trinidad and Tobago	-	NC014676	JQ228377	-	-
Tilia amurensis Kom.	Heilongjiang, China	-	NC028588	KF445432	-	-

Supplementary Table S1. Taxa and samples used in this study, geographical origin of the species, and GenBank accession numbers for cpDNA (complete plastid genome), ITS (Internal Transcribed Spacer) and NRC (Nuclear Ribosomal Cistrons). Expected accession numbers of cpDNA and NRC after submission to DRYAD (X) and data not used in this study (-) shown in the table.

Supplementary Table S2:

Species	Length (bp)	LSC (bp)	SSC (bp)	IR (bp)	GC %				Coding (num)	tRNA (num)	rRNA (num)
					Total	LSC	SSC	IR			
Dipterocarpus alatus	154,314	85,664	20,266	24,192	37.0	34.8	31.6	43.1	87	31	8
D. retusus	154,131	85,541	20,224	24,183	37.0	34.8	31.5	43.1	88	31	8
D. turbinatus	156,706	85,334	20,291	24,136	37.4	34.9	31.4	44.4	89	29	8
Hopea chinensis	151,899	84,755	19,536	23,804	37.3	35.1	31.9	43.3	88	30	8
H. hainanensis	151,684	84,666	19,512	23,747	37.3	35.2	31.9	43.3	88	31	8
H. reticulata	151,838	84,669	23,848	21,658	37.3	35.2	34.4	43.0	88	30	8
Parashorea chinensis	151,596	85,515	22,627	20,095	37.9	35.8	33.0	43.7	88	30	8
Shorea assamica	153,049	84,183	19,870	25,243	37.6	35.4	31.9	44.1	88	31	8
Vatica guangxiensis	151,033	83,290	20,003	23,870	37.2	35.2	31.3	43.2	88	30	8
V. mangachapoi	152,900	83,496	20,082	23,916	37.4	35.2	31.3	43.1	88	31	8
V. rassak	151,564	83,551	20,201	23,906	37.2	35.2	31.1	43.1	88	30	8
Abelmoschus esculentus	163,121	88,071	19,032	28,009	36.7	34.5	31.5	42.0	87	36	8
Aquilaria sinensis	159,565	87,024	19,855	26,144	37.1	35.0	31.6	42.9	87	34	8
Arabidopsis thaliana	154,478	84,170	17,780	26,264	36.3	34.0	29.3	42.3	85	37	8
Daphne kiusiana	171,491	85,028	2,681	41,891	37.7	34.8	28.3	38.9	89	38	8
Gossypium barbadense	160,317	88,841	20,294	25,591	37.2	35.2	31.7	43.0	84	37	8
Hibiscus syriacus	161,019	89,698	19,831	25,745	36.8	34.7	31.1	42.8	79	34	8
Talipariti hamabo	161,729	89,217	19,570	26,471	36.9	34.8	30.9	42.7	85	37	8
Theobroma cacao	160,619	89,333	20,194	25,546	36.9	34.7	31.2	42.9	81	37	8
Tilia amurensis	162,715	91,124	20,397	25,597	36.5	34.1	31.0	42.9	85	37	8

Supplementary Table S2. Characteristics of the plastomes used in this study. Large Single Copy (LSC) regions, Small Single Copy (SSC) regions, Inverted Repeats (IR), GC content of each region, and number of coding tRNA and rRNA loci, are shown.

Supplementary Table S3:

	Gene family	Gene
1	Photosystem I	*psaA,psaB,psaC,psaI,psaJ*
2	Photosystem II	*psbA,psbB,psbC,psbD,psbE,psbF,psbH,psbI,psbJ,psbK,psbL,psbM,psbN,psbT*
3	Cytochrome b/f complex	*petA,petB,petD,petG,petL,petN*
4	ATP synthase	*atpA,atpB,atpE,atpF,atpH,atpI*
5	NADH dehydrogenase	*ndhA,ndhB,ndhC,ndhD,ndhE,ndhG,ndhH,ndhI,ndhJ,ndhK*
6	RubisCO large subunit	*rbcL*
7	RNA polymerase	*rpoA,rpoB,rpoC1,rpoC2*
8	Ribosomal proteins (SSU)	*ps2,rps3,rps4,rps7,rps8,rps11,rps12,rps14,rps15,rps18,rps19*
9	Ribosomal proteins (LSU)	*rpl2,rpl14,rpl20,rpl23,rpl32,rpl33,rpl36*
10	Other genes	*matK,accD,ccsA,cemA*
11	Hypothetical chloroplast reading frames	*ycf1,ycf2,ycf3,ycf4,ycf15,ycf68*

Supplementary Table S3. Gene families and genes found in all plastomes used in this study.

Supplementary Table S4:

Species name	number of reads mapped to reference	sequence length (bp)	coverage
Dipterocarpus alatus	97,702	5,789	2,173
D. retusus	165,162	5,789	3,179
D. turbinatus	244,758	5,787	4,881
Hopea chinensis	134,994	5,837	2,999
H. hainanensis	72,302	5,830	1,533
H. reticulata	174,832	5,803	2,826
Parashorea chinensis	130,502	5,803	3,699
Shorea assamica	113,056	5,801	2,428
Vatica guangxiensis	45,898	5,794	997
V. mangachapoi	33,190	5,806	892
V. rassak	47,718	5,811	1,178

Supplementary Table S4. NRC dataset characteristics. Number of reads mapped to reference, sequence length (bp) and coverage for all analyzed species are shown in the table.

Supplementary Table S5:

	Repeat 1 start (location)	Repeat 2 start (location)	Size (bp)	Type	Region
Hopea hainanensis					
1	84666 (IGS *rps19- rpl2*)	137568 (*ycf15*)	14116	P	LSC, IRb
2	99143 (IGS *ycf15- trnV-GAC*)	127925 (*ndhF*)	9270	P	IRa, SSC
3	6981 (IGS *rps16- trnQ-TTG*)	7000 (IGS *rps16- trnQ-TTG*)	148	F	LSC
Hopea reticulata					
4	137568 (*ycf15*)	137508 (IGS *trnV-GAC- ycf15*)	136	P	IRa, IRb
5	107529 (IGS *trnR-GTT- trnN-ACG*)	128832 (IGS *trnN-GTT- trnR-ACG*)	121	P	SSC
6	99150 (IGS *ycf15- trnV-GAC*)	99274 (IGS *ycf15- trnV-GAC*)	120	F	IRa
Dipterocarpus retusus					
7	94241 (IGS *ycf2- ycf15*)	94265 (IGS *ycf2- ycf15*)	110	F	IRa
8	94241 (IGS *ycf2- ycf15*)	145298 (IGS *ycf15- ycf2*)	110	P	IRa, IRb
9	145298 (IGS *ycf15- ycf2*)	145322 (IGS *ycf15- ycf2*)	110	F	IRb
Dipterocarpus turbinatus					
10	94085 (*ycf2*)	145061 (*ndhB*)	110	P	IRa, IRb
11	145037 (*ndhB*)	145061 (*ndhB*)	110	F	IRb
12	87329 (IGS *rpl23- trnI-CAT*)	151815 (IGS *ycf2- trnI-CAT*)	108	P	IRa, IRb
Hopea chinensis					
13	6990 (IGS *rps16- trnQ-TTG*)	7084 (IGS *rps16- trnQ-TTG*)	90	F	LSC
14	98824 (*ycf15*)	137736 (*ycf15*)	88	P	IRa, IRb
15	86709 (IGS *rpl23- trnI-CAT*)	86729 (IGS *rpl23- trnI-CAT*)	85	F	IRa
Vatica mangachapoi					
16	85561 (IGS *rpl23- trnI-CAT*)	149409 (IGS *ycf2- trnI-CAT*)	63	P	IRa, IRb
17	149283 (IGS *ycf2- trnI-CAT*)	149409 (IGS *ycf2- trnI-CAT*)	63	F	IRb
18	58300 (*accD*)	58450 (*accD*)	57	F	LSC
Parashorea chinensis					
19	107015 (IGS *trnN-GTT- rps15*)	126760 (*ndhF*)	54	P	SSC
20	1249 (*rpl2*)	84225 (*rpl2*)	51	P	LSC
21	108023 (IGS *trnN-GTT- rps15*)	108143 (IGS *trnN-GTT- rps15*)	51	F	SSC
Vatica guangxiensis					
22	108170 (IGS *ndhF- rps15*)	108218 (IGS *ndhF- rps15*)	50	F	SSC
23	29880 (IGS *trnE-TTC- trnT-GGT*)	29972 (IGS *trnE-TTC- trnT-GGT*)	46	F	LSC
24	85229 (IGS *rpl23- trnI-CAT*)	148948 (IGS *trnI-CAT- rpl23*)	42	P	IRa, IRb
Shorea assamica					
25	42833 (IGS *psaA- ycf3*)	42833 (IGS *psaA- ycf3*)	36	P	LSC
26	69573 (*rpl18*)	69609 (*rpl18*)	34	F	LSC
27	108889 (IGS *trnR-ACG- trnN-GTT*)	108985 (IGS *trnR-ACG- trnN-GTT*)	33	F	IRa

Supplementary Table S5. Repeat sequences and their distribution found by REPuter in Dipterocarpaceae plastomes. Large Single Copy (LSC) regions, Small Single Copy (SSC) regions, Inverted Repeat A (IRa), Inverted Repeat B (IRb), IGS: Intergenic spacer. F and P indicate repeat type F (forward) and P (palindrome), respectively [for more details **Supplementary Table S5.1. Repeat sequences and their distribution found by REPuter in Dipterocarpaceae plastomes** (see Excel file)].

Supplementary Table S6:

No.	SSR type	SSR	size	start	SSR-containing region
Dipterocarpus turbinatus					
1	p1	A	12	70600	IGS (*rpl33- rps18*); LSC
4	p1	A	11	7818	IGS (*rps16- trnQ-TTG*); LSC
	p1	A	11	32847	IGS (*trnT-GGT- psbD*); LSC
Dipterocarpus retusus					
3	p1	A	10	32994	IGS (*trnT-GGT- psbD*); LSC
	p1	A	10	57308	IGS (*atpB- rbcL*); LSC
	p1	A	10	63555	*cemA*; LSC
Vatica guangxiensis					
1	p1	T	15	94332	*ndhB*; IRa
1	p1	T	13	51379	IGS (*ndhC- trnV-TAC*); LSC
1	p1	T	12	2224	*matK*; LSC
Vatica mangachapoi					
1	p1	T	11	60906	IGS (*ycf4- cemA*); LSC
2	p1	T	10	7272	IGS (*psbK- psbI*); LSC
	p1	T	10	8810	IGS (*psbK- psbI*); LSC
Hopea reticulata					
2	p1	T	10	83139	IGS (*rpl14- rps3*); LSC
	p1	T	10	125520	IGS (*rpl32- ndhF*); SSC
1	p1	C	10	1915	IGS (*psbA- matK*); LSC
Hopea chinensis					
3	p2	(AT)6	12	45634	IGS (*ycf3- trnS-GGA*); LSC
	p2	(AT)6	12	32092	IGS (*trnT-GGT- psbD*); LSC
	p2	(AT)5	10	4135	IGS (*matK- rps16*); LSC
Hopea hainanensis					
2	p2	(TA)7	14	62429	IGS (*ycf4- cemA*); LSC
	p2	(TA)6	12	1676	IGS (*psbA- matK*); LSC
1	p3	(AAC)4	12	14435	*atpL*; LSC
Parashorea chinensis					
1	p3	(AAC)4	12	14835	*atpL*; LSC
1	p3	(TTC)4	12	68219	IGS (*psaJ- rpl33*); LSC
1	p4	(GAAT)3	12	110925	IGS (*trnN-GTT- rps15*); SSC
Shorea assamica					
1	p5	(GAATT)3	15	52317	IGS (*ndhC- trnV-TAC*); LSC
1	c	(TTTA)3...(T)10...(AT)8...(T)10	208	67836	IGS (*trnP-TGG- psaJ*); LSC
1	c	(ATAG)3...(TTTA)3...(A)10	191	9235	IGS (*trnS-GCT- trnR-TCT*); LSC

Supplementary Table S6. Simple sequence repeats in Dipterocarpaceae plastomes. Large Single Copy (LSC) regions, Small Single Copy (SSC) regions, Inverted Repeat A (IRa), Inverted Repeat B (IRb), IGS: Intergenic spacer shown in the table [for more details **Supplementary Table S6.1. Simple sequence repeats in Dipterocarpaceae plastomes** (see Excel file)**]**.
